# Spatio-temporal dynamics of multimodal EEG-fNIRS signals in the loss and recovery of consciousness under sedation using midazolam and propofol

**DOI:** 10.1371/journal.pone.0187743

**Published:** 2017-11-09

**Authors:** Seul-Ki Yeom, Dong-Ok Won, Seong In Chi, Kwang-Suk Seo, Hyun Jeong Kim, Klaus-Robert Müller, Seong-Whan Lee

**Affiliations:** 1 Department of Brain and Cognitive Engineering, Korea University, Seoul, Korea; 2 Department of Dental Anesthesiology, Seoul National University Dental Hospital, Seoul, Korea; 3 Machine Learning Group, Berlin Institute of Technology, Berlin, Germany; University of Electronic Science and Technology of China, CHINA

## Abstract

On sedation motivated by the clinical needs for safety and reliability, recent studies have attempted to identify brain-specific signatures for tracking patient transition into and out of consciousness, but the differences in neurophysiological effects between 1) the sedative types and 2) the presence/absence of surgical stimulations still remain unclear. Here we used multimodal electroencephalography–functional near-infrared spectroscopy (EEG–fNIRS) measurements to observe electrical and hemodynamic responses during sedation simultaneously. Forty healthy volunteers were instructed to push the button to administer sedatives in response to auditory stimuli every 9–11 s. To generally illustrate brain activity at repetitive transition points at the loss of consciousness (LOC) and the recovery of consciousness (ROC), patient-controlled sedation was performed using two different sedatives (midazolam (MDZ) and propofol (PPF)) under two surgical conditions. Once consciousness was lost via sedatives, we observed gradually increasing EEG power at lower frequencies (<15 Hz) and decreasing power at higher frequencies (>15 Hz), as well as spatially increased EEG powers in the delta and lower alpha bands, and particularly also in the upper alpha rhythm, at the frontal and parieto-occipital areas over time. During ROC from unconsciousness, these spatio-temporal changes were reversed. Interestingly, the level of consciousness was switched on/off at significantly higher effect-site concentrations of sedatives in the brain according to the use of surgical stimuli, but the spatio-temporal EEG patterns were similar, regardless of the sedative used. We also observed sudden phase shifts in fronto-parietal connectivity at the LOC and the ROC as critical points. fNIRS measurement also revealed mild hemodynamic fluctuations. Compared with general anesthesia, our results provide insights into critical hallmarks of sedative-induced (un)consciousness, which have similar spatio-temporal EEG-fNIRS patterns regardless of the stage and the sedative used.

## Introduction

Sedation is a rapidly growing technique in anesthesia care, and has become a great alternative to general anesthesia, which is considered to induce “deeper sedation” on a continuous spectrum. It is utilized for many surgical procedures such as dental procedure, plastic and reconstructive surgery, gastrointestinal endoscopy, etc [1, 2]. It has a significantly lower costs and minimal side-effects, while recovery after sedation is much faster than general anesthesia. For these reasons, patient satisfaction is also high after conscious sedation [3, 4]. However, clinical problem (e.g., oversedation) can be caused by large discrepancies in patient history, sedative preference, institutional bias, and patient/practitioner variability [5–8]. In this respect, for safe sedation by general practitioner monitoring levels of consciousness is an important clinical issue during conscious sedation. Despite the existence of approaches for measuring neurophysiological changes in the brain under general anesthesia [9–15], no studies have yet macroscopically investigated the neurophysiological dynamics of the transition points of consciousness (commonly referred to as the loss of consciousness [LOC] and the recovery of consciousness [ROC]) at the level of sedation [9, 15, 16]. Moreover, in practical aspects, the effects between 1) the sedative type and 2) the presence/absence of surgical stimulation such as scaling treatment, are poorly understood. These are particularly crucial factors for the precise estimation of the brain state of patients under surgical condition.

Electroencephalography (EEG) and functional near-infrared spectroscopy (fNIRS) are neuroimaging modalities used to monitor the brain state or to establish direct communication between the human brain and machine [17–21]. These techniques are greatly valued for their ability to track the brain state under general anesthesia [9, 10, 22, 23]. EEG recordings have typically been used in studies on consciousness due to their high temporal resolution, non-invasiveness, and portability [12]. Previous studies using EEG during general anesthesia have reported significant differences between the conscious and unconscious states. These changes include an increase in the frontal EEG power [9, 10, 15], shifts in the EEG power from high- to low-frequency bands [9, 24], and changes in the functional/effective connectivity across brain regions [25, 26]. In addition, numerous EEG-derived commercial products are commonly used in medicine for monitoring the depth of anesthesia [27–29]. Conversely, recent reports have indicated that fNIRS measurements can also efficiently track the brain state for evaluating the depth of of anesthesia by measuring hemodynamic changes in the cerebral cortex [22, 23]. fNIRS is an optical neuroimaging modality that measures the concentration changes in oxygenated (HbO) and deoxygenated (HbR) hemoglobin concentrations on the superficial layers of the human cortex. Based on previous studies, it is known that the concentration of HbO increases after focal activation of the cortex due to increased blood flow; conversely, HbR is washed out, and its levels are decreased [30–32]. Therefore, measurements obtained from NIRS are comparable to those obtained using blood oxygenation level-dependent contrast in fMRI. Furthermore, due to its relatively low cost, portability, and non-invasive nature, fNIRS technology is gathering increasing interest from the neuroscientific and brain-computer interface communities. In comparison with fMRI, fNIRS is more practical for assessing cortical activation in clinical settings [33]. Based on previous neurophysiological studies, they showed that anesthetics such as sevoflurane and propofol (PPF) inhibit neuronal activity as well as cerebral metabolism [34, 35].However, despite the active use of EEG and fNIRS for monitoring the level of consciousness, multimodal EEG/fNIRS dynamics during the LOC and ROC under sedation remain poorly understood for practical use in clinical procedures.

Here we hypothesized the following. Around transition points (LOC and ROC) during sedation, there would be similar spatio-temporal EEG spectral patterns between two surgical conditions (i.e., non-scaling stage without surgical stimulation vs. dental-scaling stage with surgical stimulation) regardless of the sedative used, but have significant differences in the level of effect-site concentrations (CEs) in the brain that are required for loss and recovery of consciousness. Furthermore, we hypothesized that critical points in multimodal EEG–fNIRS dynamics would be associated with the transition states of consciousness (i.e., LOC and ROC) during sedation; these could be used as gold standard references for consciousness under sedation. To verify our hypotheses, we simultaneously recorded EEG and fNIRS measurements during a non-scaling procedure and thereafter during a dental scaling procedure with surgical stimulation under the patient-controlled sedation (PCS) [36]. In PCS, patients self-administer the sedative. This sedation technique is more suitable than computer-controlled sedation because the patients can comfortably and safely maintain a proper and minimal level of consciousness, allowing us to observe the transition points at LOC and ROC, repetitively.

In the study, we analyzed the spatio-temporal EEG and fNIRS measurements together with CEs to evaluate the abovementioned hypotheses for both surgical stages (non-scaling vs. dental scaling) and sedatives (midazolam (MDZ) vs. PPF). Our study revealed that EEG and fNIRS dynamics are neurophysiological correlates of consciousness under sedation, and that they may reveal novel characteristics allowing monitoring of the level of consciousness even during sedation.

## Material and methods

### Subjects and clinical procedures

Sixty normal healthy volunteers (15 females; ASA Physical Status 1 or 2; mean age 26.87 ± 5.37 years) participated in our experiments. Subjects were devided into groups of 10 according to the type of sedative (MDZ and PPF) and its bolus doses (low, middle, and high). We calculated the sample size (= 10) of 3 dose groups for each sedative based on analysis of variance (ANOVA) with the difference in the maximum infused count of 3, the standard deviation of 1.8, *α* of 0.05 and *β* of 0.8, and 10% drop-out rate according to the previous work on PCS-based comparison analysis [37]. In this study, we selected 40 normal healthy volunteers receiving middle and high bolus doses (10 females; mean age 27.3 ± 6.3 years). Subjects who received a low bolus dose were excluded due to lack of data on the LOC and ROC. Both MDZ and PPF treatment groups comprised 20 subjects (5 females). We used a randomized double-blinded protocol. Two male subjects in the MDZ group were excluded due to insufficient artifact-free EEG data caused by frequent head movements throughout the experiment. Thus, data from 38 participants were used. Our experiment was approved by the Institutional Review Board of the Seoul National University Dental Hospital and was registered with the clinical research information service (CRiS), Republic of Korea (URL: https://cris.nih.go.kr/cris/index.jsp). The registration number is KCT0001618. The study was conducted in accordance with the Declaration of Helsinki. None of the subjects had a history of cardiovascular or respiratory disease (including asthma), head trauma or surgery, neurological/psychiatric disorder, or previous problems associated with the administration of anesthesia. All volunteers gave their informed written consent and received financial compensation for their inconvenience.

Prior to sedation, subjects were required to fast for at least 3 h and 6 h in terms of their liquid and solid intake, respectively. For safety purposes, each subject was monitored using standard intraoperative management (BM7, Bionet, Seoul, Korea) (capnography, non-invasive blood pressure, pulse oximetry, and electrocardiography) and bispectral index (BIS) measurements (BIS^™^, Covidien, MA). For standardization, the experiment was performed by three supervisors, including an anesthesiologist and an identical dentist. We also recorded videos to evaluate each subject’s behavior at different levels of sedation in the study. After the end of the experiment, each subject and dentist reported the pain intensity of the dental treatment using a 5-point Likert scale.

### Experimental design and protocols

**Fig 1:**
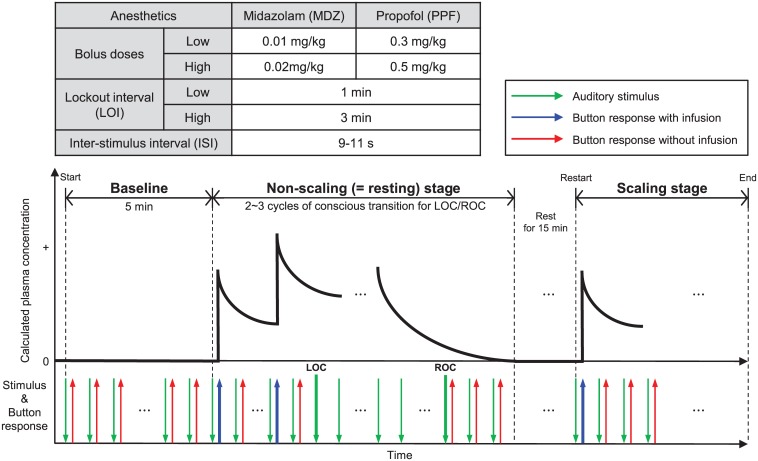
Experimental designs. Protocols for both anesthetics for patient-controlled sedation (top), experimental timeline with the calculated plasma concentration, stimulus onset, and button-response sites during electroencephalography and functional near-infrared spectroscopy measurement recordings (bottom).

Sedation was performed via patient-controlled intravenous infusion of MDZ or PPF (Perfusor^®^ space syringe pump system, B. Braun Medical Inc., Melsungen, Germany) with two different bolus doses and lockout times (Fig 1). The self-administration device was programmed to deliver middle or high doses of MDZ or PPF. Subjects in the middle-dose group were infused with 0.01 mg/kg of MDZ or 0.3 mg/kg of PPF at a speed of 1500 ml/h, and with a lockout interval of 1 min. Subjects in the high-dose group were infused with 0.02 mg/kg of MDZ or 0.5 mg/kg of PPF, with a lockout interval of 3 min. The sedative was administered through a three-way stopcock and a 22-G intravenous catheter inserted into a vein of the left hand. Subjects were instructed to lie on a dental chair with their eyes closed to avoid artifacts caused by eye movement throughout the experiment. Subjects were also asked to hold the self-administration button of the infuser in their right hand to respond to the prerecorded auditory stimulus of “Press the button”, followed by a beeping sound. The auditory stimulus as a command was presented every 9–11 s at random to avoid the effect of the subject predicting the timing of the next auditory stimulus. Subjects were guided to press the self-administration button if they heard the auditory stimuli in their headphones. This led to the infusion of the sedative into their veins. Therefore, sedative could be injected according to the subject’s response to auditory stimuli and lockout times only when subjects were conscious. The subjects were trained to wait until the end of the stimulus before responding to eliminate the effects of auditory event-related potentials. The experimental procedure was divided into two sequential phases: the non-scaling and dental-scaling phases. During the dental-scaling phase, the subjects had their teeth scaled by a dentist to remove dental calculus using an ultrasonic scaler, and the subjects were only under sedation during the non-scaling phase. For the dental scaling phase, a low intensity of pain at approximately 15–19 on a visual analog scale, which is a reliable and valid pain measurement scale for evaluation of dental pain, was produced by dental ultrasonic scaling [38–41]. The sedative infusion was discontinued 15 min prior to the end of each scaling phase. There was a 15-min interval between the two phases to facilitate preparation for the scaling procedure and allow the subjects to rest.

### Data acquisition

**Fig 2:**
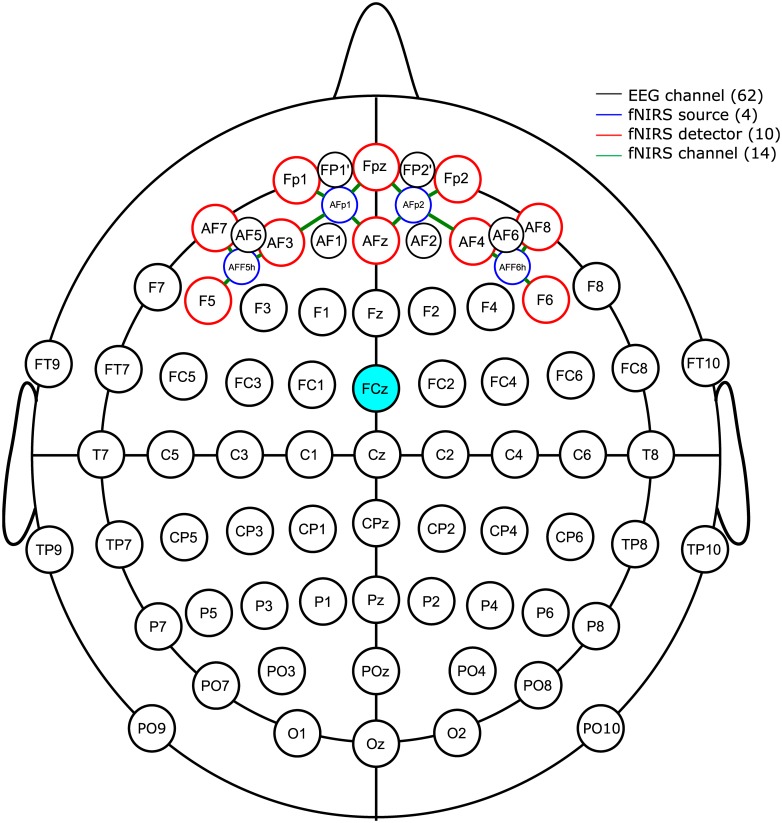
The selected EEG-fNIRS channel locations of the International 10–20 system. The selected electroencephalogram (EEG) and functional near infrared spectroscopy (fNIRS) channel locations of the International 10–20 system (62 EEG recording electrodes (black circle), 4 fNIRS sources (blue circle), 10 fNIRS detectors (red circle), and 14 fNIRS channels (green line).

During the study, simultaneous EEG and fNIRS measurements were performed. EEGs were recorded using a sampling rate of 1 kHz with a multichannel EEG amplifier (BrainAmp, Brain Products, Munich, Germany) from 62 Ag/AgCl electrodes on a cap (64Ch-actiCAP, Brain Products) according to the International 10–20 system. Impedances of the EEG electrodes were reduced to below 10 *k*Ω prior to data collection. EEG recordings were down-sampled to 100 Hz using a 10th-order digital Chebyshev filter before analysis. Furthermore, the reference electrode standardization technique was utilized to re-reference scalp EEG recordings by standardizing the reference electrode to a point at infinity [42]. On the other hand, we used an NIRS-System (NIRScout 8–16, NIRx Medizintechnik GmbH, Berlin, Germany) equipped with 24 optical fibers (4 sources with wavelengths of 760 nm and 850 nm and 10 detectors convolving to 14 measurement channels) covering the frontal areas of the head. HbO and HbR concentration changes were calculated using the modified Beer–Lambert law [43, 44]. The sampling frequency for the fNIRS was 15.625 Hz. To use BIS as well as the EEG and fNIRS systems simultaneously, for safety purposes, BIS electrodes were placed onto the forehead under the EEG and fNIRS channel locations to prevent contact with fNIRS light sources and detectors. EEG electrodes and fNIRS probes were integrated into a standard EEG cap with inter-optode distances of 2–3 cm. The optical probes are constructed such that they fit into the ring of standard electrodes. This enabled us to situate the NIRS channel positions according to the International 10–20 system. The locations of the fNIRS and EEG channels are shown in Fig 2.

### Data analysis

To analyze EEG and fNIRS dynamics, we first defined behavioral time markers for each subject based on the auditory-response task. We used these markers to compare and pool data across subjects. We defined the LOC as the time point at which the subject responded to the auditory stimuli for at least the previous 5 times and subsequently stopped responding for at least the next 10 times. The ROC was defined as the time point at which the subject did not respond to the auditory stimuli for at least 10 previous presentations and continually responded to the stimuli for at least the next 5 times. To analyze spatio-spectral dynamics using data from the signals, EEG data epochs were extracted for every subject with reference to previous work, as follows [9]: (1) baseline, 5 min prior to induction; (2) consciousness → unconsciousness, -1 to 3 min with respect to LOC; (3) unconsciousness → consciousness, -3 to 1 min with respect to ROC; and (4) recovery, 4 min prior to the end of resting phase. Therefore, spatio-temporal EEG and fNIRS analyses are based on segmented epochs with a dimension of 62 channels × 24000 time samples for EEG and 12 channels × 62 time samples for fNIRS, respectively. On average, subjects lost and recovered consciousness 1.7–2.5 times for each sedative (MDZ, 2.1 and 1.7; PPF, 2.5 and 2.0 in non-scaling and scaling stages, respectively). EEG signals were divided into several frequency bands that included delta (0.1–3 Hz), theta (4–7 Hz), lower alpha (8–12 Hz), upper alpha (12–15 Hz), and beta/gamma (15–40 Hz). EEG data analysis was performed using EEGLAB 13.4.4b (Delorme and Makeig, 2004; http://sccn.ucsd.edu/eeglab/) running under MATLAB^™^ R2012a (MathWorks, Natick, MA).

During the non-scaling stage for both sedatives, fNIRS signals were divided into consciousness → unconsciousness (-4 to 6 min with respect to LOC) or unconsciousness → consciousness (-6 to 4 min with respect to ROC). We also extracted fNIRS signals during the baseline period for 3 min to perform subject-level baseline-correction. We then calculated the centered moving average with a window size of 1.5 min using the Berlin Brain-Computer Interface (BBCI) (github.com/bbci/bbci_public) toolbox [45]. The fNIRS data in the scaling stage were excluded due to significant artifacts, such as frequent head movements or inevitable contacts by the dentist during the scaling procedure.

Furthermore, to calculate CEs which allow an estimation of what happens to the administered MDZ and PPF in the brain for each subject, two different pharmacokinetic and pharmacodynamic models were used in calculation of the plasma concentration and CE for MDZ [46, 47] and PPF [48, 49], respectively.

### Spectral analysis

Analyses of event-related changes in spectral power across single trials, time-locked to experimental events, can characterize event-related perturbations in the oscillatory dynamics of ongoing EEG signals [50]. Therefore, we computed ERSPs using sinusoidal wavelet-based time-frequency decomposition to measure event-locked changes in spectral power. We computed subject-level baseline subtractions for induction and emergence with respect to the LOC and ROC by subtracting the baseline mean spectrum for each subject. For *n* trials, *F*_*k*_(*f*, *t*) of ERSP is the spectral estimate of trial *k* at frequency *f* and time *t* as follows,

$$\begin{array}{c} \text{ERSP}\left(f,t\right)=\frac{1}{n}\sum_{k=1}^{n}{\left|F_{k}\left(f,t\right)\right|}^{2} \end{array}$$ (1)

Frequencies were extracted between 0 and 40 Hz, and the number of wavelet cycles and the padding ratio were both 3, with increasing cycles and 1, during linear cycles. Group ERSPs were calculated as log power values in dB by aggregating data from the five frontal channels (AF3–4, F1–2, and Fz) across all subjects. Scalp EEG plots were generated using the “scalpplot” function in the BBCI toolbox. To evaluate event-related spectral changes around the transition of consciousness quantitively with respect to anesthetics and stages, statistical significant differences in ERSPs were assessed using t-tests with the null hypothesis of equal means.

### Functional connectivity analysis

Functional connectivity between fronto-parietal areas (five frontal areas: F1–4 and Fz and five parietal areas: PO3–4, POz, Pz, and Oz) was assessed using event-related phase coherence (ERPCOH) with a phase delay. Thus, we determined the degree of synchronization between the two activity measures in different sets of trials. In EEGLAB, for two signals, *a* and *b*, ERPCOH is defined using the following equation:

$$\begin{array}{c} {\text{ERPCOH}}^{a,b}\left(f,t\right)=\frac{1}{n}\sum_{k=1}^{n}\frac{F_{k}^{a}\left(f,t\right)F_{k}^{b}{\left(f,t\right)}^{*}}{|F_{k}^{a}\left(f,t\right)F_{k}^{b}\left(f,t\right)|} \end{array}$$ (2)

$F_{k}^{a}{\left(f,t\right)}^{*}$ is the complex conjugate of $F_{k}^{a}\left(f,t\right)$. The instantaneous phase difference between two EEG time series ranges from −180° to 180°.

The instantaneous phase difference is the arctangent of the imaginary part of ERPCOH divided by its real part at each time point [51, 52]. We then calculated a moving-average and used Savitzky-Golay filtering of the ERPCOH using a window size of 20 s (non-overlapping) to smooth out short-term fluctuations and highlight longer-term trends or cycles during transitions of consciousness.

## Results

### Spatio-temporal EEG dynamics

**Fig 3:**
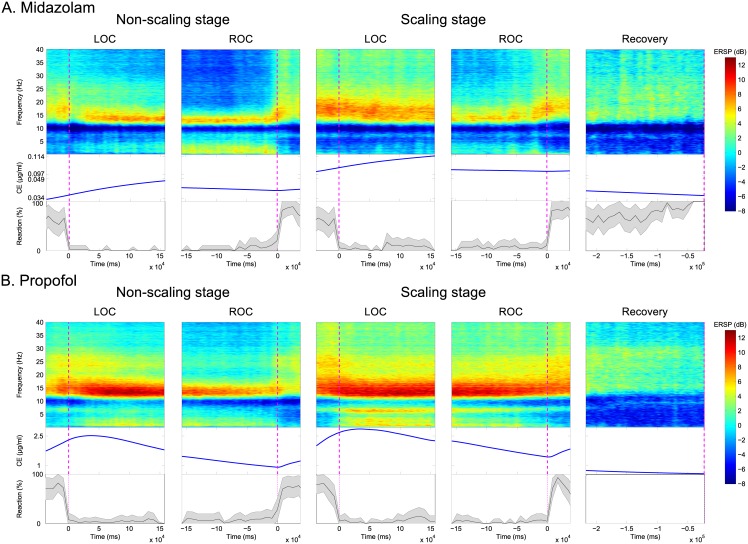
Dynamics of baseline-normalized time-series event-related spectral perturbations (ERSPs) from the five frontal channels (top layer), the corresponding effect-site concentrations (CEs) in the brain (middle layer), and the time-course of reaction curves for auditory stimuli (bottom layer) aligned with respect to the loss of consciousness (LOC) (1^st^ and 3^rd^), recovery of consciousness (ROC) (2^nd^ and 4^th^), and recovery (5^th^ column) phases according to the stage using (A) midazolam and (B) propofol. Magenta vertical lines denote the transition time-points of the LOC, ROC, and recovery.

**Fig 4:**
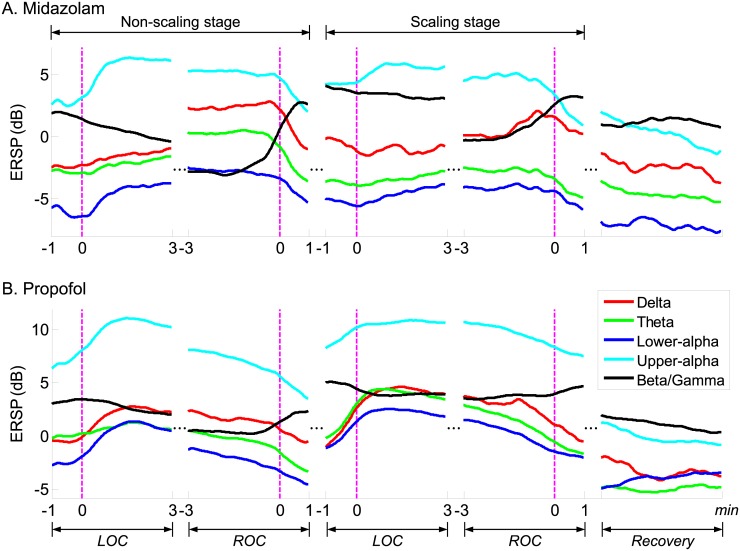
Time-series event-related spectral perturbation (ERSP) dynamics aligned with the loss of consciousness (LOC), recovery of consciousness (ROC), and recovery phases averaged in five frequency ranges (delta, theta, lower/upper alpha, and beta) within two stages. Magenta vertical lines denote the transition time-points of the LOC, ROC, and recovery.

For quantitative observation of the spectral EEG dynamics around the LOC and ROC during sedation, we used event-related spectral perturbations (ERSPs) in the spatio-temporal EEG domain during two stages (non-scaling vs. dental scaling) and with two sedatives (MDZ vs. PPF) [50]. We performed subject-level baseline-subtraction for the LOC, ROC, and recovery by subtracting the mean of the baseline spectrum for each subject. To reduce spatial blur distortion of the EEG signal, we then averaged the EEG channels from five frontal areas (AF3–4, F1–2, and Fz) that are strongly associated with the depth of anesthesia according to previous studies [10] (Fig 3 and S1 Fig for an individual subject). In addition, CEs in the brain were calculated to allow comparison of time-series EEG patterns during sedation according to the different types of sedatives, as well as the different stages. Moreover, behavioral response curves were calculated as the speed of response, indicated by time-lapse between the the button press and the auditory stimulus for behavioral evaluation of the level of consciousness throughout the experiment. We then generated moving-average EEG spectrograms of group-level time courses for five predefined frequency bands to compare frequency-wise EEG oscillations in the spatio-temporal domain (Fig 4).

For both stages, during MDZ-induced sedation, we observed increased EEG spectral dynamics in the delta, theta, lower alpha, and upper alpha frequency bands, whereas beta/gamma power was decreased during the LOC interval. There was a marked increase in the upper alpha power. During the non-scaling stage, we observed rising and falling curves with steeper slopes at the LOC point (i.e., time = 0) compared with those observed during the scaling stage (Figs 3A and 4A). During the ROC interval, these EEG spectral dynamics in ERSP occurred in reverse. ERSP patterns clearly decreased in the aforementioned four frequency bands, whereas spectral power in the beta/gamma band increased. Dynamic changes in ERSP around the ROC point for each frequency band were rapidly shifted during both stages. During the final recovery, spectral EEG dynamics in all frequency bands slowly reverted to the spectral powers observed at baseline (Fig 4). Time-series ERSP powers in the beta/gamma band were, on average, maintained at relatively higher levels during the scaling stage in both the LOC and ROC intervals. On the other hand, CEs of MDZ gradually increased for both intervals, even until 3 min after the LOC, and were slightly reduced until the ROC following delivery of the next bolus injection when patients pressed the button.

For both stages, EEG spectral patterns obtained with PPF-induced sedation were broadly in line with those observed under MDZ-induced sedation for the delta, theta, lower alpha, highlighted upper alpha, and beta/gamma frequency bands during the LOC interval, although the EEG spectral power was relatively higher than that observed with MDZ. For both stages, the rising and falling curves also had steep slopes from the LOC point until 1 or 2 min after the LOC for each subject (Figs 3B and 4B). During the ROC interval, in contrast to the pattern observed for the LOC, we noted gradually increasing and decreasing EEG spectral patterns in the selected frequency bands. In addition, the EEG spectral powers in all frequency bands slowly converged to the spectral powers observed at baseline during the recovery. During both stages, CEs of PPF increased until immediately after the LOC and then gradually diminished until the subjects recovered consciousness. Due to the fast recovery from PPF-induced sedation, the LOC and ROC intervals overlapped in some subjects. During the scaling stage, PPF also had higher CEs in the brain. After the ROC, the CE of PPF increased again due to the next bolus injection of PPF. A comparison of the ERSP spectral powers between PPF and MDZ revealed a greater enhancement of power in every frequency range for PPF-induced sedation than for MDZ-induced sedation. For both sedatives, when transitioning through consciousness evels, the changes in the upper alpha power were the most significant.

**Fig 5:**
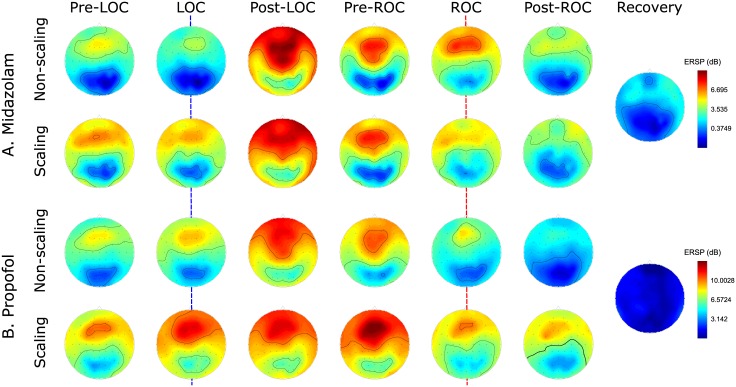
Spatial distribution of the event-related spectral perturbation (ERSP) in the upper alpha band at different points during the loss of consciousness (LOC), recovery of consciousness (ROC), and recovery phases according to the stage using (A) midazolam and (B) propofol. The pre-LOC/ROC was the first 10 s of time intervals for the LOC/ROC, the LOC/ROC was -5 to +5 seconds aligned with transition points, the post-LOC/ROC was the last 10 s of time intervals for the LOC/ROC, and the recovery was the last 10 s before full recovery.

To represent the spatial distribution of EEG changes over time, we included topographical maps of the highlighted upper alpha frequency in predefined time intervals in Fig 5 and S2 Fig for for an individual subject. We selected specific time intervals based on the LOC, ROC, and recovery points as follows: 1) pre-LOC/ROC, first 10 s during the LOC/ROC; 2) LOC/ROC, -5–5 s relative to the LOC/ROC; 3) post-LOC/ROC, last 10 s during the LOC/ROC; and 4) recovery, last 10 s of the experiment. We found that, with a change in the level of consciousness, most but not all spatial EEG patterns in the frontal and parieto-occipital EEG channels changed markedly. During both MDZ and PPF-induced sedation stages, most frontal areas were clearly activated during unconsciousness (post-LOC and pre-ROC) and were deactivated after the ROC. During the scaling stage, spatial EEG patterns were similar to those observed during the non-scaling stage, whereas ERSP at the pre-LOC was higher. For both sedatives, significant differences in spectral power were observed at time-points corresponding to the induction and emergence of consciousness. When comparing the two stages in the pre-LOC, the spectral power in the frontal area was relatively enhanced during the scaling stage. Table 1 summarizes statistical tests between frontal EEG spectral powers within time intervals of consciousness and unconsciousness (i.e. Pre-LOC vs. Post-LOC and Pre-ROC vs. Post-ROC) in every frequency range according to the stages and sedatives used. When we compared level of consciousness around the LOC and ROC for each sedative and stage, a paired *t*-test revealed that EEG spectral powers were significantly changed in almost every time interval. Additionally, EEG spectral dynamics in the upper alpha band were highly significant, regardless of the type of sedative and the stage.

**Table 1:** Statistical comparison of frequency-wise event-related spectral changes around the transition of consciousness with respect to anesthetics and stages. *, **, and *** indicate the level of significant improvement with *p* < 0.05, *p* < 0.01, and *p* < 0.001, respectively. *p*-values are based on paired t-tests.

Type	MDZ				PPF			
	Non-scaling stage		Scaling stage		Non-scaling stage		Scaling stage	
	Pre- vs. Post-LOC	Pre- vs. Post-ROC	Pre- vs. Post-LOC	Pre- vs. Post-ROC	Pre- vs. Post-LOC	Pre- vs. Post-ROC	Pre- vs. Post-LOC	Pre- vs. Post-ROC
Delta	2.542*	-3.546***	-6.857	-0.212	2.745**	-3.103**	6.971***	-6.857***
Theta	3.755***	-9.836***	-8.884	-2.833**	1.192	-7.813***	5.161***	-8.884***
Lower-alpha	3.940***	-6.222***	-9.140	-2.618*	6.447***	-4.346***	6.880***	-9.140***
Upper-alpha	6.940***	-6.340***	-6.054**	-4.271***	6.886***	-6.276***	5.577***	-6.054***
Beta/Gamma	-5.835***	14.514***	3.368*	6.046***	-1.800	4.695***	-2.392*	3.368**

**Fig 6:**
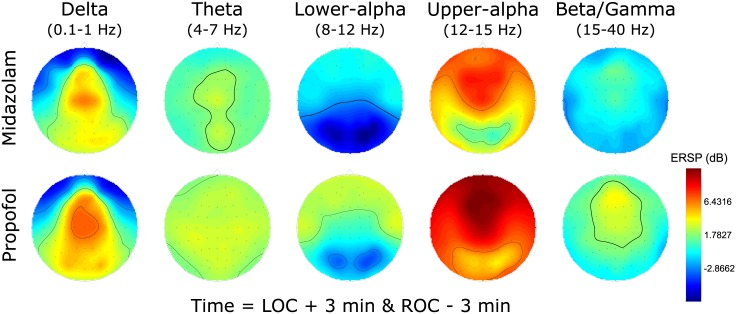
Differences between conscious and unconscious sedation in the spatial EEG distribution of power for the frequency bands. We selected time-points for unconscious sedation = loss of consciousness (LOC) + 3 min and recovery of consciousness (ROC)—3 min.

**Fig 7:**
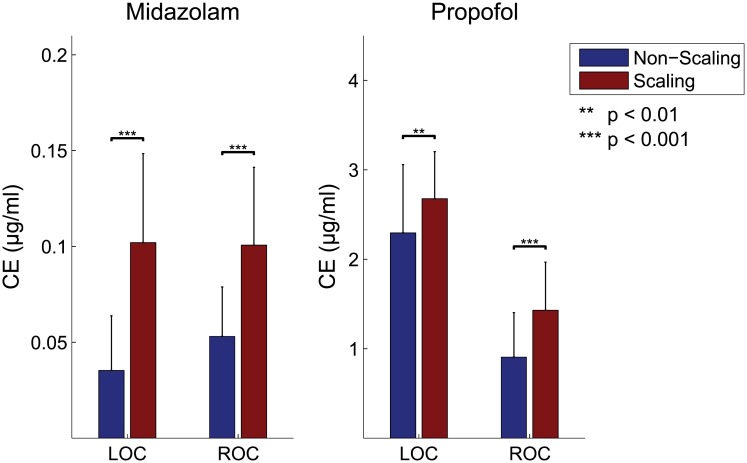
Statistical comparison of effect-site concentrations (CEs) in the brain between the different types of conditions for midazolam (left) and propofol (right) during the loss and recovery of consciousness.

When performing frequency-wise spatio-spectral comparisons between the baseline and unconscious states (during the post-LOC and pre-ROC), the spatial distributions in the pre-frontal and parieto-occipital areas differed for delta, in the parieto-occipital area for lower alpha, and in the temporal and frontal area for upper alpha rhythms, for both sedatives (Fig 6). EEG spectral powers were higher during PPF sedation than during MDZ sedation, particularly in the frontal area for lower alpha, and in the occipital area for upper alpha rhythms. We used a subject-level paired *t*-test to compare CEs at the LOC and ROC (Fig 7). For both sedatives, the difference in CEs between stages was statistically significant. This result illustrates that on average, the CEs for MDZ were approximately 2-fold higher during the non-scaling stage than those during the scaling stage, whereas the CEs for PPF were smaller but significantly different between the two stages at both the LOC and ROC (MDZ, *p* < 0.001 at the LOC and *p* < 0.001 at the ROC; PPF, *p* < 0.01 at the LOC and *p* < 0.001 at the ROC). With MDZ, most subjects lost and recovered consciousness with similar CEs, whereas with PPF, most subjects recovered consciousness (during the ROC) with CEs lower than those observed during the LOC for both stages.

### Functional connectivity by EEG

**Fig 8:**
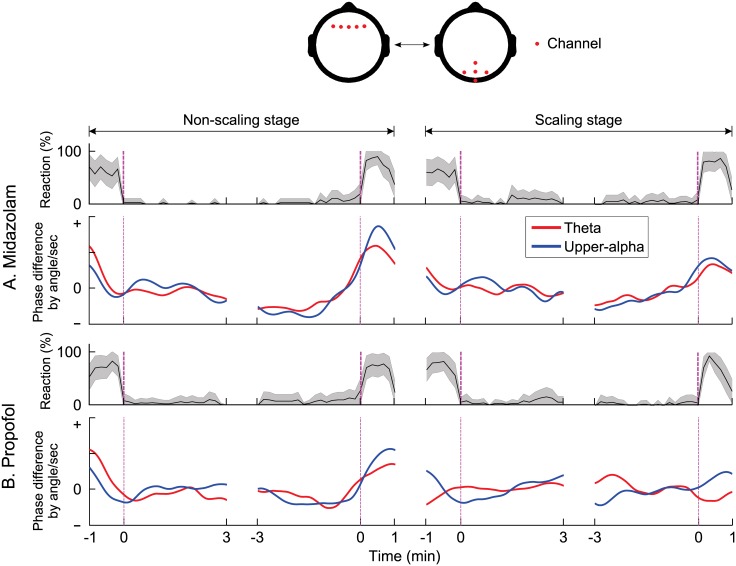
Grand-averaged time-courses of reaction curves for auditory stimuli (top layer) and group-level phase difference in the time domain with 5 × 5 fronto-parietal paired channels and two frequency ranges (theta and upper alpha) (bottom layer) for (A) midazolam and (B) propofol. For group-level phase difference, the x-axis indicates the time intervals of the loss and recovery of consciousness during the two stages, and the y-axis was calculated with a time-series of instantaneous phase differences between all pair-wise combinations of fronto-parietal channels to show the phase relationship between channels [51, 52].

To examine functional connectivity from paired channels that were associated with consciousness, we computed group-level ERPCOH with five frontal (F1–4 and Fz) and five parieto-occipital (PO3–4, POz, Pz, and Oz) EEG channels at specific frequency bands (theta and upper alpha). In addition, we adopted a technique to assess instantaneous EEG phase differences to clearly reveal the direction of the phase information between the paired EEG channel groups over time (Fig 8 and S3 Fig for an individual subject). We found critical points approximately before the LOC and after the ROC. These points were more noticeable during the non-scaling stage for both sedatives, as this stage had fewer artifacts caused by head movement or by the scaling treatment. During the non-scaling stage, there were clear sudden negative- and positive-directed phase shifts on the right before the LOC and after the ROC, respectively. Thereafter, a prolonged period of phase stability was observed following a phase shift for both sedatives. During unconsciousness (LOC interval: 0–3 min and ROC interval: -3–0 min) due to MDZ sedation, we observed more steady phase differences between fronto-parietal channel groups than those during consciousness. With PPF sedation, there were frequent and slight phase shifts.

### Spatio-temporal fNIRS dynamics

**Fig 9:**
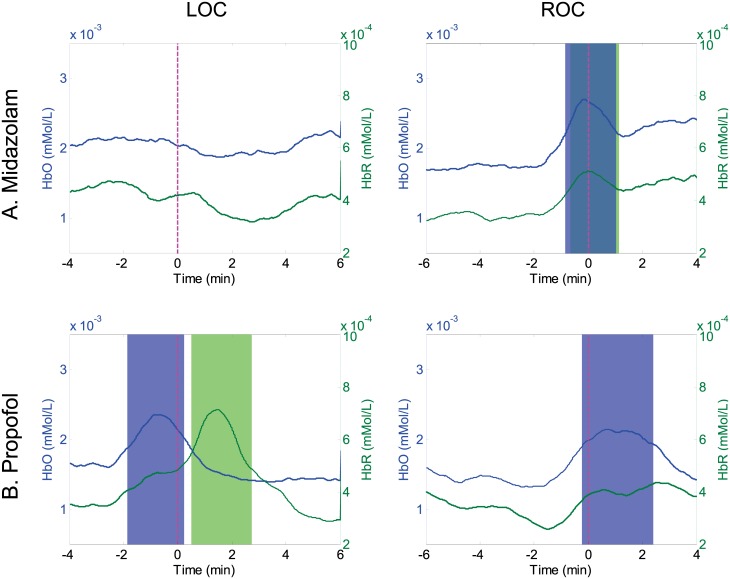
Time course oxygenated (HbO) and deoxygenated (HbR) hemoglobin measurements during the loss of consciousness (LOC) and recovery of consciousness (ROC) for (A) midazolam and (B) propofol administration during the non-scaling stage. Each shaded area indicates the selected time intervals with the statistical significance (Blue: HbO, Green: HbR). Vertical lines denote the transition points of the LOC and ROC.

Fig 9 displays the grand averaged time courses of HbO and HbR measurements during the LOC and ROC for both sedatives across 14 fNIRS channels. Together with spatio-temporal EEG dynamics, the fNIRS analysis also revealed some critical points with statistically significantly increased peaks as compared to those observed at baseline. MDZ-induced hemodynamic changes exhibited no significant time intervals during the LOC. In contrast, during the ROC, statistically significantly different time intervals of -1–1 min appeared at both HbO and HbR measurements. At 2 min before the ROC, both HbO and HbR gradually increased until the transition point into consciousness (0 s) and then decreased again. With PPF, unlike with MDZ, significant time intervals of HbO and HbR sequentially appeared during the LOC. An increment peak of HbO was observed at approximately -2–0 min, and thereafter a significant peak in HbR measurement was observed. With PPF, no peak was observed for the HbO time intervals after the ROC.

## Concluding discussion

Research on consciousness using drug-induced general anesthesia or sedation has major clinical significance and has been conducted using diverse experimental protocols to date. Previous studies mainly focused on the level of consciousness under the effect of general anesthesia. Few studies have investigated the effects of neural dynamics during the LOC or ROC under sedation by means of EEG or fNIRS. Furthermore, to our knowledge, no studies have compared EEG neural dynamics according to the different types of sedatives as well as surgical conditions. Therefore, we macroscopically investigated brain activity using spatio-temporal EEG and fNIRS as well as the dynamic changes in CEs in the brain at both the LOC and ROC in subjects sedated with different sedatives (MDZ or PPF) during surgical conditions. We repetitively induced the LOC and ROC while maintaining the level of sedation around the transition point of consciousness. We observed a correlation between the upper alpha rhythm of the EEG and sedation, and identified critical points in the EEG and fNIRS measurements, i.e., a sudden phase transition between fronto-parietal areas and mildly increased fNIRS signatures during the LOC and ROC.

### Neural correlates of EEG and fNIRS during (Un)consciousness

Our findings are based on sedation, and they are consistent with the results of previous work performed under general anesthesia and sleep conditions. Both sedatives lead to similar spatio-temporal EEG patterns. We reported that during the transition into and out of unconsciousness, both MDZ and PPF lead to an increase in ERSP powers in almost all frequency ranges, excluding that of the beta/gamma band. The highest ERSP power was concentrated in the upper alpha band (Figs 3 and 4). These EEG spectral dynamics are considered to have similar effects on the synchronization of thalamo-cortical interactions, which were observed in a study on PPF-induced general anesthesia [53]. During increases in the dose of a sedative in the brain, intracortical synchrony may block the hyperpolarization of neurons in the thalamic relay nuclei from propagating specific signals upward and may induce LOC [26]. The highest upper alpha power that we observed is an identifying feature of non-rapid eye movement sleep and is believed to mediate many sleep-related functions ranging from memory consolidation to cortical development, as observed in studies on sleep [54, 55].

These phenomena suggest that the electrophysiological mechanism underlying sedation is shared with those of sleep as well as general anesthesia. In addition, our results agree with those reporting general anesthesia-based EEG signatures, which have recently been termed traveling peaks. These signatures are coalescent continua of the coherent oscillation from the beta and gamma range to the alpha range during the transition into unconsciousness, and they indicate that sedation occurs on an identical continuum, moving through moderate sedation to deep sedation [9, 56]. Therefore, we postulate that a coherent frequency range can be used as a hallmark of the depth of consciousness under general anesthesia and sleep, as well as sedation. Our study generated more frequent LOCs and ROCs at lighter sedative levels than those generated using general anesthesia; however, these coalescence effects across frequency ranges were equivalent for both sedatives. Therefore, during both MDZ- and PPF-induced LOC, a similar mechanism may act via the *γ*-aminobutyric acid (GABA)-mediated inhibition of neuronal firing [57]. Previous work revealed that under equal sedation levels, PPF produces an equivalent degree of memory impairment as that produced by MDZ [58].

Spatial dynamics in EEG upper alpha power are symmetric during the LOC and ROC after sedation due to a characteristic process referred to as anteriorization [59, 60] (see Fig 5). We found that during rest (baseline), when the subject’s eyes are closed, upper alpha activity is predominantly observed in the occipital area. As noted during the induction of LOC, the anteriorization of power increases from the beta bands to the upper alpha range for pre- and post-LOC. During the ROC, we observed a reversed decrease in the frontal upper alpha power. An abrupt transition from posterior to anterior upper alpha power occurs during LOC [53]. This anteriorization effect is reflected by increased GABA inhibition induced by both sedatives, resulting in a differential effect on thalamic nuclei with disparate spatial projections [59]. These results support the concept that spatial dynamics between anterior and posterior cortices may also be hallmarks of consciousness under sedation as well as during general anesthesia or sleep. Furthermore, our comparison of frequency-wise spatial distributions during unconsciousness with those during wakefulness indicates that sedation is generally accompanied by a spatially distributed increase in the parieto-occipital delta and frontal upper alpha powers and a decrease in the parieto-occipital lower alpha power; this is in agreement with previous reports (Fig 6) [9, 10, 61].

Comparisons of drug-dependent spatial distributions under MDZ or PPF sedation indicates that their EEG patterns are similar in almost all frequency ranges and that there are only small differences in the lower and upper alpha of frontal and parietal area. This occurs despite the fact that the two sedatives induce different levels of sedation. Concurrent EEG- and fNIRS-based multi-modal analyses for both sedatives indicate that the time-series dynamics of CEs in the brain are pharmacodynamically altered depending on the type of sedative (Fig 3). For both sedatives and during both stages, CEs have positive patterns during the LOC and negative patterns until the ROC. Based on the pharmacodynamics model that we adopted, we estimated that the CE of PPF increases until 2–3 min after the LOC and is slightly decreased until the ROC, at which point the subject is again induced by sedative [62]. We found different CEs during the LOC and ROC depending on the type of sedative used (Fig 7). Although there was no significant difference between the two CEs during MDZ-induced sedation, subjects that were sedated using PPF recovered their consciousness at relatively lower CEs than those observed upon the LOC. Anesthetics such as MDZ, PPF, and diazepam suppress long-term potentiation in the hippocampus and lead to the inhibition of cerebral metabolism. Our results indicate that hemodynamic responses from fNIRS may be linked to disruption of brain functional integration. Previous studies have reported a significant increase in HbO levels in response to PPF induction [22, 63]. However, in the present study, due to the limitation of fNIRS electrode placement around parieto-occipital brain area during scaling treatment, spatial processing of fNIRS and/or coupling analysis between modalities (i.e. EEG vs. fNIRS) are absent. Therefore, these issues should be investigated further in future.

Previous neuroimaging studies have reported that altered states of consciousness are associated with a breakdown of dominating fronto-parietal feedback connectivity, as assessed by EEG and fNIRS recordings under general anesthesia, in a vegetative state, or during sleep [16, 25, 64–66]. Fluctuations in network connectivity over time occur because of changes in the levels of vigilance, task switching, or conscious processing [67–69]. However, the spectral and temporal dynamics in EEG activity and connectivity underlying sedative-induced LOC and ROC are poorly understood. Therefore, we computed dynamic feedback connectivity using time-series EEG data over fronto-parietal channels, encompassing the anterior and posterior cingulate cortex regions, which may represent thalamo-cortical interactions, as reported previously [25, 70]. These data were used to detect changes in cortical information transfer during the LOC and ROC induced by two different classes of sedatives. We found that during MDZ/PPF-induced LOC and ROC, abrupt phase shifts in fronto-parietal connectivity occurred within significantly short time intervals (Fig 8).

The abrupt changes in the directionality of fronto-parietal connectivity are consistent with other findings, suggesting that consciousness results from the integration of global neural information, whereas unconsciousness is caused by disrupting in the capacity for this integration, and is linked to the disruption of dominant feedback communication in the fronto-parietal network [71]. This change in directional connection has been reported in the stduies of animal under general anesthesia and appears transiently after the administration of a PPF bolus in humans [72, 73]. This finding may be a result of a significant disruption in phase synchronization between the frontal and parietal regions [72, 74] as well as disruption of optimal functional networks in the parietal region [75]. Therefore, the fact that directional connectivity of EEG phase dynamics is non-stationary may play an important role in determining the depth of sedation, because it exhibits characteristic changes during sedation [16, 73, 76, 77].

In comparison with general anesthesia and sleep, the results of our spatio-temporal EEG and fNIRS dynamics indicate that although the level of consciousness is broadly divided into sleep, sedation, and general anesthesia, these stages share mechanisms in some functional areas. However, we postulate that the effect of sedation is closer to that of sleep than that of general anesthesia given the pronounced frontal upper alpha EEG spectral patterns observed during sedation.

### Comparisons of EEG with and without surgical stimulation

We also compared stages with and without surgical stimulation (i.e., scaling treatment). We wanted to study the relationship between EEG and CE dynamics with and without noxious stimulation. We found similar positive or negative ERSP patterns with both sedatives, although the ERSP patterns had different CEs. We also observed statistically significant differences in CEs between the two stages during the LOC and ROC (Fig 7). Brain CE data obtained through pharmacodynamic analysis indicated that CEs were approximately 2-fold higher during the scaling treatment for both sedatives. This was consistent with the previous finding that a higher CE of the sedative is required to achieve a desired sedation level and induce a given EEG effect in clinical settings that included surgical stimulation [78]. However, the EEG patterns of neural dynamics showed a similar change in almost every frequency range, independent of surgical stimulation. Although unavoidable events around the head region during scaling treatment may result in artifacts leading to a low signal-to-noise ratio, we were still able to observe a significant EEG pattern based on a spectral analysis.

## Supporting information

S1 Fig Dynamics of baseline-normalized time-series event-related spectral perturbations (ERSPs) from the five frontal channels for individual subject (4 of (A) midazolam and (B) propofol). Throughout the experiment, blue and red vertical lines denote the transition time-points of the LOC and the ROC for each subject, respectively.
(PDF)

S2 Fig Individual spatial distribution of the event-related spectral perturbations (ERSPs) in the upper alpha band at different points during the loss of consciousness (LOC), recovery of consciousness (ROC), and recovery phases according to the stage using (A) midazolam and (B) propofol. (PDF)

S3 Fig Subject-wise time-courses of phase differences in the time domain with 5 × 5 fronto-parietal paired channels for two frequency ranges (theta and upper alpha) for several subjects, with (A) midazolam- and (B) propofol-induced sedation. (PDF)
