# Supplementary figures and images for: Spatio-temporal dynamics of multimodal EEG-fNIRS signals in the loss and recovery of consciousness under sedation using midazolam and propofol
Source: PLoS One. 2017 Nov 9;12(11):e0187743. doi: 10.1371/journal.pone.0187743 (PMC5679575; doi:10.1371/journal.pone.0187743)

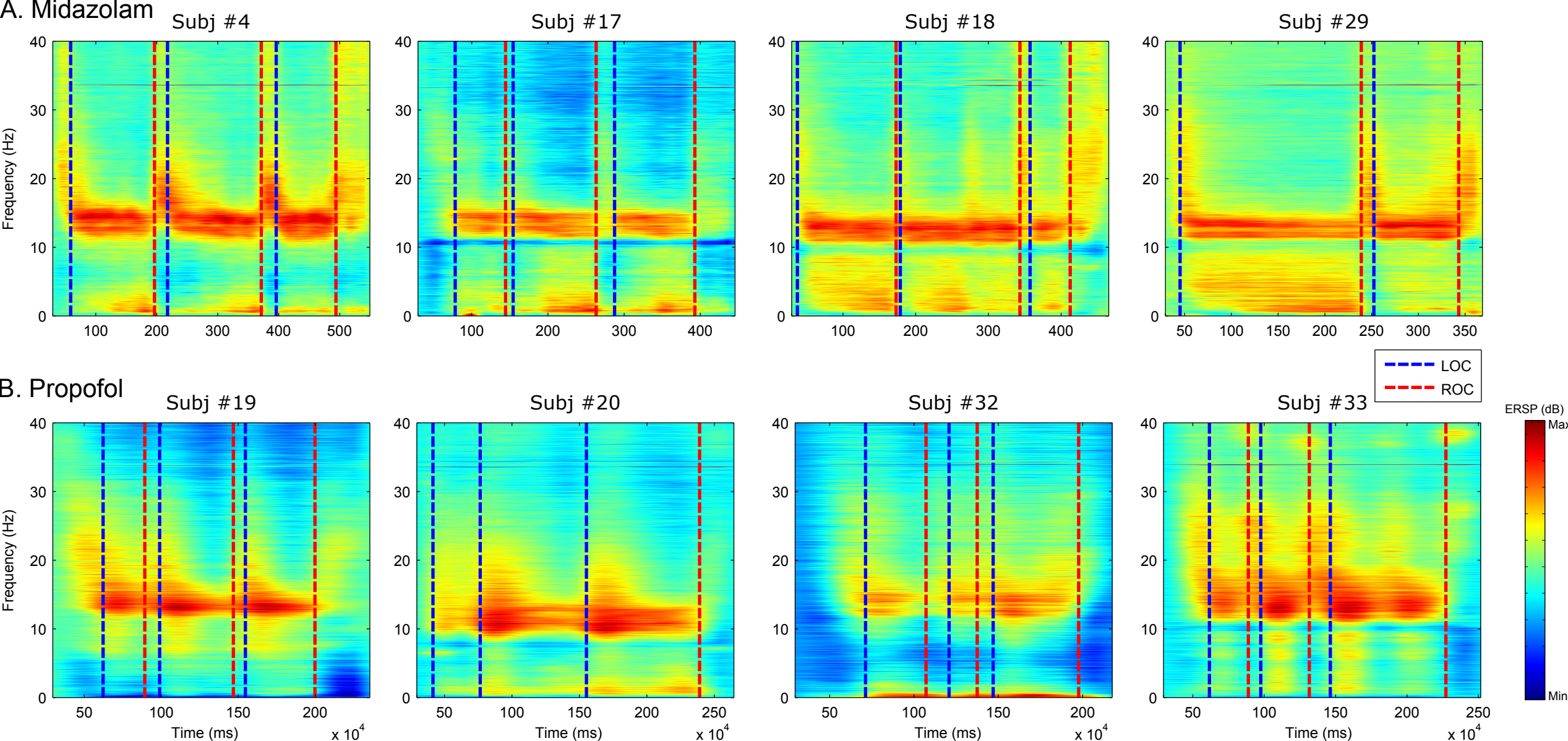

Supplement: S1 Fig — Throughout the experiment, blue and red vertical lines denote the transition time-points of the LOC and the ROC for each subject, respectively. (PDF) [file pone.0187743.s001.pdf]

# A. Midazololam

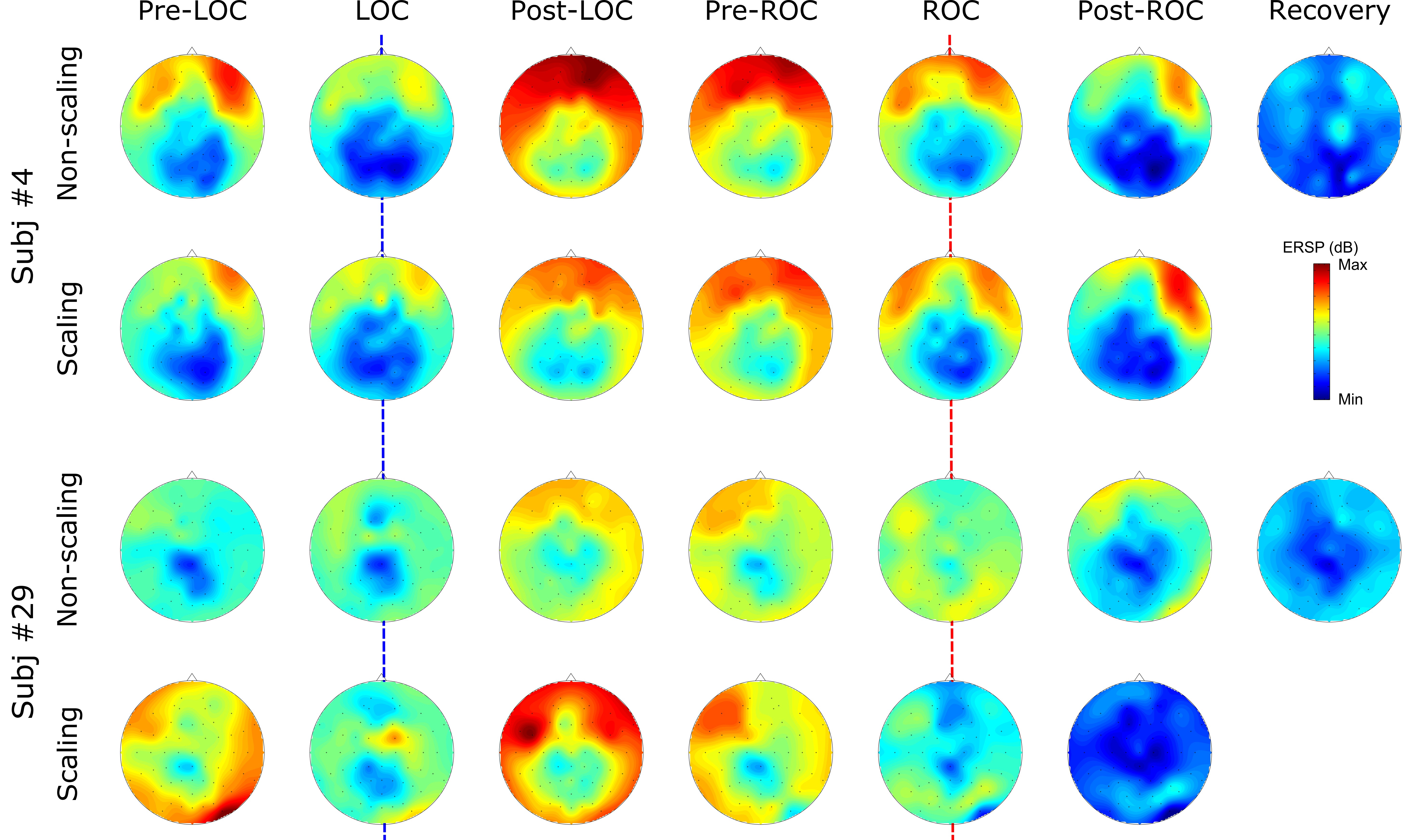

# B. Propofol

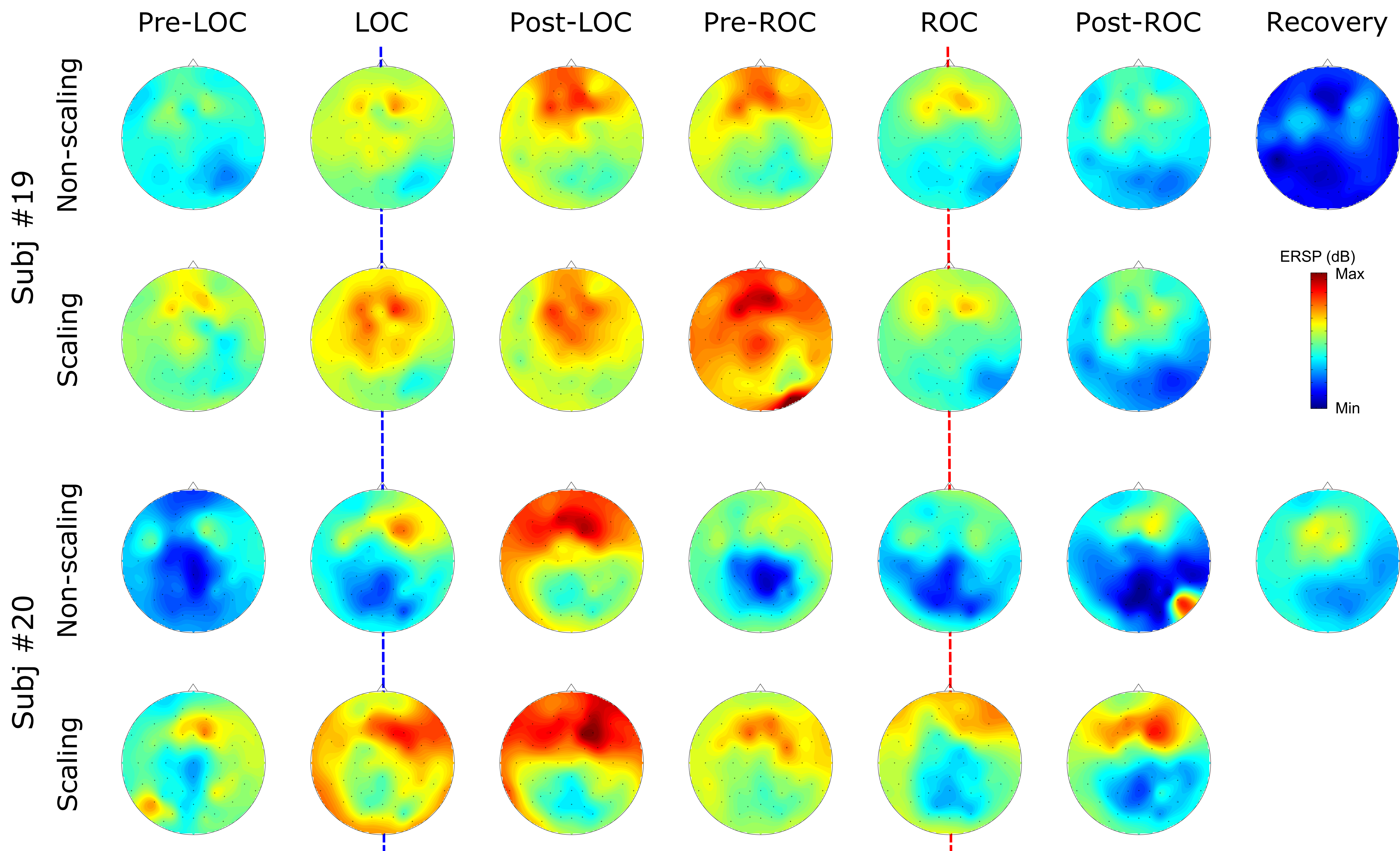

Supplement: S2 Fig — (PDF) [file pone.0187743.s002.pdf]

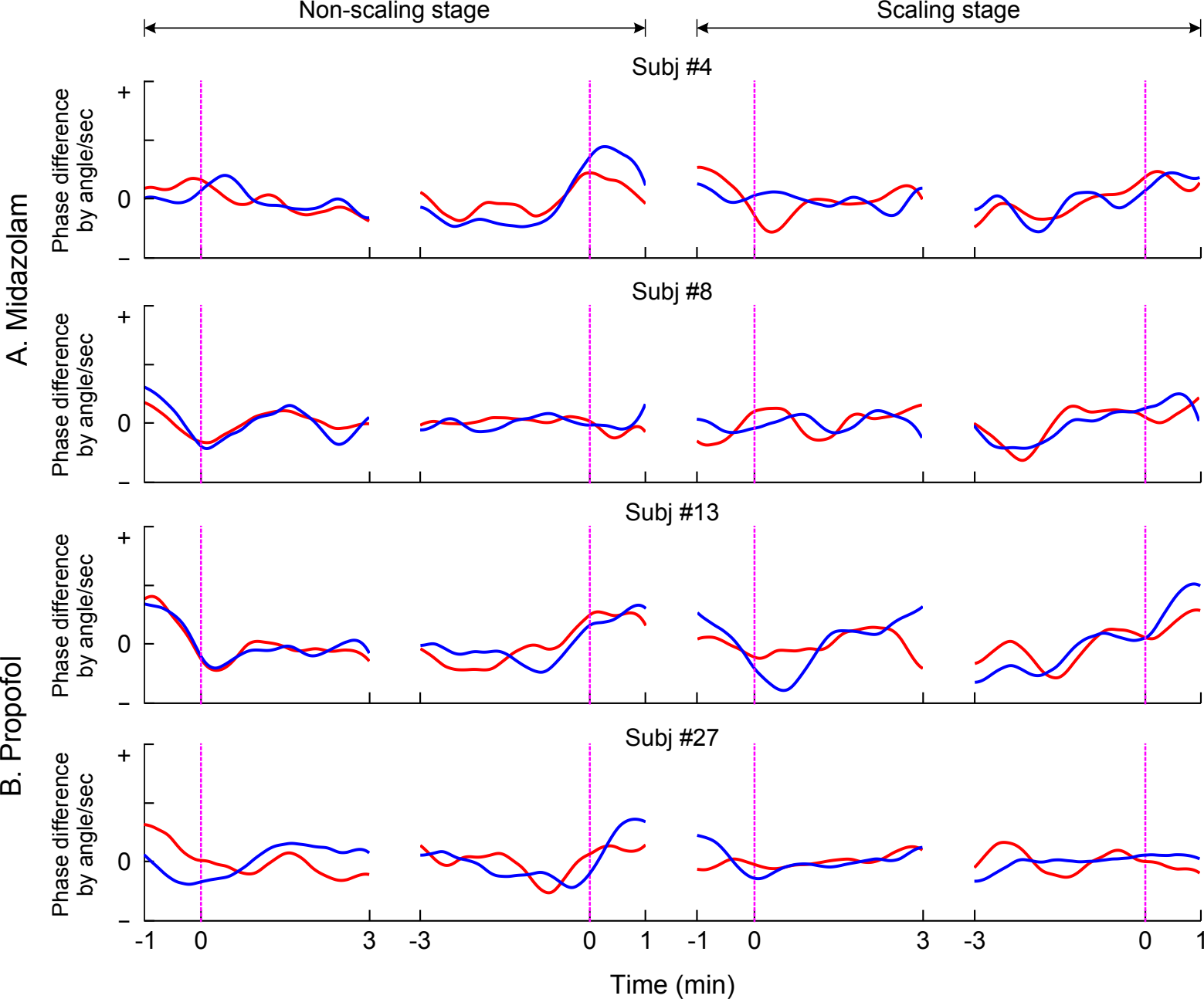

Supplement: S3 Fig — (PDF) [file pone.0187743.s003.pdf]
